# Establishment of primary and immortalized fibroblasts reveals resistance to cytotoxic agents and loss of necroptosis-inducing ability in long-lived Damaraland mole-rats

**DOI:** 10.1007/s11357-024-01420-9

**Published:** 2024-12-02

**Authors:** Yusuke Suzuki, Kanta Yamaguchi, Kaitlyn N. Lewis Hardell, Kurumi Ota, Taira Kamikado, Yoshimi Kawamura, Rochelle Buffenstein, Kaori Oka, Kyoko Miura

**Affiliations:** 1 Department of Aging and Longevity Research, Kumamoto University, 2-2-1 Honjo, Chuo-Ku, Kumamoto, 860-0811 Japan; 2 Calico Life Sciences LLC, South San Francisco, USA; 3 Department of Biological Sciences, University of Illinois, Chicago, USA; 4 Center for Metabolic Regulation of Healthy Aging, Kumamoto University, Kumamoto, 860-8556 Japan

**Keywords:** Damaraland mole-rat, Fibroblast, Cell culture, Immortalized cell, Toxin resistance, Necroptosis

## Abstract

The Damaraland mole-rat (DMR; *Fukomys damarensis*) is a long-lived (~ 20 years) Bathyergid rodent that diverged 26 million years ago from its close relative, the naked mole-rat (NMR). While the properties of NMR cultured fibroblasts have been extensively studied and have revealed several unusual features of this cancer-resistant, long-lived species, comparative DMR studies are extremely limited. We optimized conditions for successfully culturing primary DMR skin fibroblasts and also established immortalized DMR cells using simian virus 40 early region expression. Like NMRs, DMR fibroblasts are more resistant than mice to various cytotoxins including heavy metals, DNA-damaging agents, oxidative stressors, and proteasome inhibitors. DMR genome sequencing analyses revealed the presence of premature stop codons in the master regulator genes of necroptosis, an inflammatory programmed cell death—receptor-interacting protein kinase 3 (*RIPK3*) and mixed lineage kinase domain-like (*MLKL*), although these mutations have different locations to those found in the NMR. DMR cells, like NMR cells, did not show significantly increased cell death in response to necroptosis induction. Our data suggest that both Bathyergid species require species-specific cell culture conditions for optimized growth, display similar resistance to cytotoxins, and show loss-of-function mutations abrogating the ability to employ necroptosis. These shared traits may contribute to their evolved adaptations to their subterranean lifestyle and prolonged longevity. These convergent insights and valuable resource may be pertinent to biomedical research.

## Introduction

**Fig. 1:**
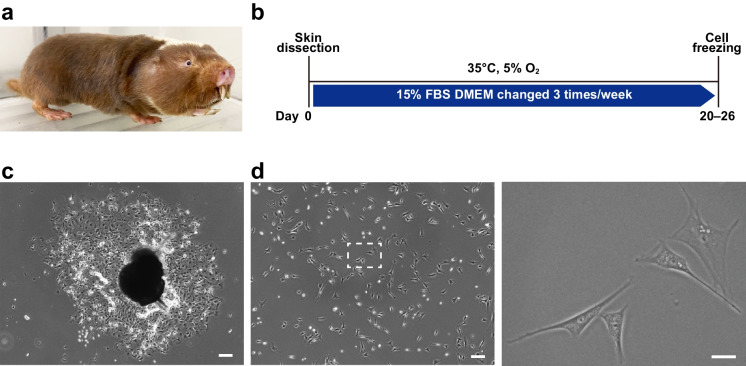
Isolation and culture of fibroblasts from Damaraland mole-rat (DMR) skin. **a** A DMR. **b** Timeline of isolation and culture of DMR fibroblasts. **c** Image of a piece of skin and the surrounding proliferating cells after 9 days of culture. Scale bar: 200 µm. **d** Primary skin fibroblasts from a DMR. The boxed region in the left image is enlarged in the right image. Scale bars: 200 µm (left) and 50 µm (right)

The Damaraland mole-rat (DMR, *Fukomys Damarensis*, Fig. 1a) is a eusocial, long-lived, medium-sized mole-rat species that inhabits a complex maze of underground tunnels in the arid and semi-arid regions of southern and central Africa [1]. DMRs live in social groups of up to 40 individuals and show a pronounced reproductive skew, limiting reproduction to a single breeding female within the social group [2, 3]. Like other subterranean rodents, both captive and free-living DMRs have a lower metabolic rate than expected on the basis of body size [4–8] as well as a lower, albeit seasonally variable, body temperature [9] than observed in small mammals living above ground. Living below ground presents with multiple physiological challenges [10]. Here both heat and gas exchange are limited because of sealed, dank, poorly ventilated burrows exacerbated by soil properties that may restrict diffusion of gases. DMRs are extremely tolerant of bouts of hypoxia [11, 12]. Their milieu also is devoid of exposure to UV radiation, and not surprisingly, DMRs are chronically vitamin D deficient [13] yet nevertheless are able to well-maintain mineral homeostasis [14]. Aside from these ecophysiological adaptations [10] many of which are shared with the extraordinarily long-lived, cancer resistant, naked mole-rat (*Heterocephalus glaber*, NMR) [15], little is known about DMR biology [16–18].

Despite their relatively small body size (average size 142 g for females and 165 g for males) [3], the 20-year maximum lifespan of DMRs in captivity is twice that predicted on the basis of body size [19, 20] and is approximately half as long as that observed in its close relative, the NMR from which it diverged 26 million years ago [21]. The NMR is now accepted as a burgeoning non-traditional model for biomedical research [22, 23]. It is also regarded as a counter example to the inevitability of age-related decline or demise based upon its defiance of Gompertzian rules of mortality [20, 24], age-associated well-maintained body composition [25], metabolic rate, and heart function [26, 27], and their resistance to chronic age-associated diseases such as cancer, neurodegeneration, and cardiovascular disease [23, 28]. Little is known if the DMR shares these traits and indeed, until recently the DMR has been overlooked in biomedical research [17], with most publications restricted to their ecology and physiology [17, 29–33].

DMRs, like the NMR, do not show a typical exponential increase in mortality risk with advancing age, but show a modest linear increase in risk of dying as they get older [20, 24, 26]. Similar to the NMR, they show no age-related decline in the basal metabolic rate. The cultured fibroblasts from both species show a greater dependence on glycolysis rather than oxidative phosphorylation for ATP production [6, 25]. Mitochondria from DMR and NMR hearts show a lower production of hydrogen peroxide relative to shorter-lived rodents [34]. However, this did not appear to translate to reduced oxidative damage or enhanced antioxidative defense [35, 36]. A recent study showed that DMRs produce high-molecular-mass hyaluronan like NMRs [37].

Although genome sequencing of the DMR has identified several candidates for potential longevity-related gene alterations, e.g., *PRDX2*, *PRDX5*, *FASTK*, and *IGF2* [21], the molecular mechanisms underlying the characteristics of DMRs remain poorly understood. Tremendous insights into the extraordinary, biomedically relevant phenotypes of NMRs have been obtained largely using in vitro cultured cells of NMRs. For example, studies using cultured NMR fibroblasts have reported various cellular and molecular mechanisms that likely contribute to resistance of NMRs to cancer and aging, such as early contact inhibition by secretion of high-molecular-mass hyaluronan [38], resistance or vulnerability to various types of stressors [39], a cell death-inducing mechanism through serotonin metabolism in senescent cells [40], high translational fidelity [41], more efficient DNA repair [42, 43], and an inability to induce necroptosis, a programmed necrotic cell death that induces tissue inflammation and is associated with several age-related diseases [44]. Considering the similarities and differences between DMRs and NMRs, it is important to investigate the characteristics of DMR fibroblasts and compare them with NMR fibroblasts in order to understand the convergent and divergent evolutionary processes involved in their longevity, delayed aging, and cancer resistance. Nevertheless, studies of cultured DMR fibroblasts remain very limited and optimization of the culture conditions has not been reported.

In this study, we isolated primary fibroblasts from DMR skin and optimized the culture conditions. In addition, we established immortalized DMR fibroblasts using simian virus 40 early region (SV40ER). Using these cells, we revealed that DMRs, similar to NMRs [39, 44], are more resistant than mice to a broad spectrum of cytotoxic stressors. Moreover, DMRs have likely lost their necroptosis-inducing ability due to loss-of-function mutations in the receptor-interacting protein kinase 3 (*RIPK3*) and mixed lineage kinase domain-like (*MLKL*) genes, which are the master regulators of necroptosis.

## Materials and methods

### Animals

DMRs were maintained at both Kumamoto University Japan and Calico LLC, USA. All DMRs (1–5 years old) used in this research were raised in a room that was maintained at 25 ± 1 °C and 50 ± 10% humidity with a 12-h light and 12-h dark cycle. All experiments involving animals were in accordance with the Guide for the Care and Use of Laboratory Animals (US National Institutes of Health). The Ethics Committees of Kumamoto University approved all procedures (approval No. A2020-042 and A2022-079) as did the IACUC committee of Calico Life Sciences (B-2–2020; B-9–2020).

### Isolation of primary skin fibroblasts

DMR fibroblasts were isolated from the back skin of adult DMRs aged 1–5 years. DMR skin was collected under anesthesia using a combination of anesthetic agents including 0.3 mg/kg medetomidine (Dorbene, Kyoritsu Seiyaku), 4 mg/kg midazolam (Dormicum, Maruishi Pharmaceutical), and 5 mg/kg butorphanol (Vetorphale, Meiji Animal Health). Skin was washed with ice-cold phosphate-buffered saline (PBS, Nacalai Tesque) containing 0.6% glucose (Nacalai Tesque or FUJIFILM, WAKO), 1% penicillin/streptomycin (Nacalai Tesque), 12.5 µg/mL amphotericin B (FUJIFILM, WAKO), and 100 µg/mL gentamicin (Nacalai Tesque). The skin was cut into small pieces and resuspended in Dulbecco’s Modified Eagle Medium (DMEM; Sigma-Aldrich or FUJIFILM, WAKO) containing 15% fetal bovine serum (FBS, NICHIREI), 2 mM L-glutamine (FUJIFILM, WAKO), 1% penicillin/streptomycin, 1% MEM Non-essential Amino Acids (FUJIFILM, WAKO), 12.5 µg/mL amphotericin B, and 100 µg/mL gentamicin. The skin pieces were seeded onto 10-cm gelatin-coated dishes (IWAKI) and incubated at 32 °C or 35 °C in a humidified atmosphere of 5% CO_2_ and 5% O_2_. The medium was replaced with new medium three times per week. Primary mouse skin fibroblasts were obtained from the back skin of 6–8-week-old C57BL/6N mice [45].

### Cell culture

Primary DMR or mouse skin fibroblasts were used up to passage ten. Primary fibroblasts prepared from skin tissues of at least three animals were used as biological replicates. The cells were cultured in DMEM containing 15% FBS, 2 mM L-glutamine, 1% penicillin/streptomycin, and 1% MEM Non-essential Amino Acids at 32 °C, 35 °C, or 37 °C in a humidified atmosphere of 5% CO_2_ and 5% O_2_ or at 32 °C in 5% CO_2_ and 3%, 5%, or 21% O_2_. The medium was replaced with new medium every 2 days. Cell culture was performed in an incubator or Hypoxia Chamber (STEMCELL Technologies) under the respective atmospheric conditions. Briefly, the chamber was filled with mixed gas consisting of nitrogen, O_2_, and CO_2_ at appropriate levels, sealed, and placed in an incubator.

### Cell growth analysis

DMR primary fibroblasts were seeded at a density of 1 × 10^5^ cells in 6-cm dishes (Thermo Fisher Scientific) or 3 × 10^5^ cells in 10-cm dishes (Greiner Bio-One) and grown to sub-confluency. DMR SVER cells were seeded at a density of 1.5 × 10^5^ cells in 6-cm dishes and grown to sub-confluency. Sub-confluent cells were trypsinized, counted using a Countess 3 FL Automated Cell Counter (Thermo Fisher Scientific), and reseeded on a new dish.

### Cell death analysis

DMR fibroblasts were seeded at a density of 1 × 10^4^ cells per well in 4-well plates (Thermo Fisher Scientific). At sub-confluency, cells were stained with Hoechst 33342 (Dojindo, 1 µg/mL in growth medium) in the incubator for 10 min and then with propidium iodide (PI; FUJIFILM, WAKO; 5 µg/mL in growth medium) in the incubator for 5 min. Images were acquired with a BZ-X 810 fluorescence microscope (Keyence) and positive cells were counted with a BZ-X Image Analyzer (Keyence). In total, 4–6 random microscope fields (at least 50 cells) per cell line were analyzed. Hoechst- and PI-double-positive cells were counted as dead cells.

### Cellular senescence analysis

DMR fibroblasts were seeded at a density of 1 × 10^4^ cells per well in 4-well plates. At sub-confluency, cells were fixed in PBS containing 2% formaldehyde (MUTO PURE CHEMICALS) and 0.2% glutaraldehyde (Nacalai Tesque) for 10 min at room temperature, washed twice with PBS, and stained with SA-β-Gal staining solution (details below) at 37 °C for 2 days and then with Hoechst 33342 (1 µg/mL in PBS) at room temperature for 30 min. SA-β-Gal staining solution contained 5 mM potassium hexacyanoferrate(III) (Nacalai Tesque), 5 mM potassium hexacyanoferrate(II) trihydrate (Nacalai Tesque), 2 mM magnesium chloride (Sigma-Aldrich), 150 mM sodium chloride (Nacalai Tesque), 36.9 mM citric acid (FUJIFILM, WAKO), 126.2 mM disodium hydrogen phosphate (FUJIFILM, WAKO), and 1 mg/mL 5-bromo-4-chloro-3-indolyl-β-D-galactopyranoside (Nacalai Tesque). Images were acquired with a BZ-X 810 fluorescence microscope, and Hoechst-positive cells were counted using a BZ-X Image Analyzer. SA-β-Gal-positive cells were counted manually. Four random microscope fields (at least 80 cells) per cell line were analyzed. Hoechst- and SA-β-Gal-double-positive cells were counted as senescent cells.

### Lentiviral transduction of SV40ER

Lentiviruses were prepared as previously described using the pCSII-EF-SV40ER-TK-hyg vector [the backbone vector (pCSII-EF-Rfa-TK-hyg) was kindly provided by Dr. Hayato Naka-Kaneda (Shiga University of Medical Science, Japan)] [44, 46]. DMR fibroblasts were transduced with the lentiviral vector expressing SV40ER. Four days after transduction, cells were cultured in medium containing 250 µg/mL hygromycin (FUJIFILM, WAKO) for 8–10 days. Subsequently, hygromycin-resistant cells (DMR SVER cells) were established.

### Cytotoxic insults

For cytotoxicity assays, 1 × 10^4^ fibroblasts between passages 5 and 10 were seeded/well of a 96-well plate in triplicate 24 h prior to xenobiotic exposure. Mouse, NMR, and DMR primary fibroblasts were both cultured in 1% MEM with 10% FBS and 1% antibiotic–antimycotic solution at 5% CO_2_ and 3% O_2_ for the duration of toxin exposure. Cells of each of the species were housed at their documented preferred resting body temperatures, namely 37 °C, 32 °C, and 35 °C, respectively. Prior to the addition of toxic compounds, wells were washed two times with sterile PBS and fresh media were added. Compounds were dissolved in either DMSO or sterile PBS prior to the addition to the cells. After 24 h of exposure, cell viability was measured using a CellTiter-Glo assay (Promega, G7573). Absorbance from untreated controls was set to 1.0; absorbance from those samples treated with different doses of toxin was then calculated as the relative viability.

### *RIPK3* and *MLKL* sequencing analysis

DMR skin was lysed in cell lysis buffer (details below) at 55 °C overnight. Genomic DNA was extracted using phenol/chloroform/isoamyl alcohol (NIPPON GENE) and precipitated using 2-propanol (Nacalai Tesque). Cell lysis buffer contained 50 mM Tris (Sigma-Aldrich), 100 mM sodium chloride (Nacalai Tesque), 20 mM disodium dihydrogen ethylenediaminetetraacetate dihydrate (Nacalai Tesque), and 1% sodium lauryl sulfate (Nacalai Tesque). Predicted mutated regions of *RIPK3* and *MLKL* [47] were amplified by PCR with PrimeSTAR Max polymerase (TAKARA). The PCR products were purified using a QIAquick Gel Extraction Kit (Qiagen), sequenced using a SupreDye v3.1 Cycle Sequencing Kit (EdgeBio), and analyzed using an Applied Biosystems 3130 Genetic Analyzer (Applied Biosystems). The primers used for PCR and sequencing analysis are provided in Table 1.

**Table 1:** Primers used for PCR, sequencing, and qPCR

Gene/primer name	Primer	Sequence (5′–3′)
*RIPK3* (genome)	Forward	TCACAGTCAGGAACAGGGTG
	Reverse	GGAAGGGTGACAGAAAAGGC
*MLKL* (genome)	Forward	CAACTTGGACATCTCCTGGC
	Reverse	CACAAACCCAGGCTGAGATC
*ACTB*	Forward	AGACCTTCAACACCCCAGCCATG
	Reverse	GGCCAGCCAGGTCCAGACGCAG
*SV40ER*	Forward	CCTTTATAAGTAGGCATAACAGT
	Reverse	CAAATATTCCTTATTAACCCCTT
*RIPK3*	Forward	ACATTTCAGGGCAAGTCACA
	Reverse	CTGTAGACATCACTGGCCTTG
*MLKL*	Forward	CACACAAACACAGACGTTTTAC
	Reverse	TTTCAAGTCCACTTCTAACCAC
*Il6* (mouse)	Forward	TCTATACCACTTCACAAGTCGGA
	Reverse	GAATTGCCATTGCACAACTCTTT
*IL6* (DMR)	Forward	CTGCTTCACAAGCCTTTCGAC
	Reverse	CCTCTACGCAATTGGCATTATG
*TNFRSF1A*/*Tnfrsf1a* (DMR and mouse)	Forward	CAATGGGGGAGTGAGAGGC
	Reverse	CTTTGTGGCACTTGGTGCAG
*Tnfrsf1b* (mouse)	Forward	CAAGACCTCGGACACCGTGTGTGC
	Reverse	GCCTGCACACATCAGTGG
*TNFRSF1B* (DMR)	Forward	CAAGACCTCAGACACTGTGTGT
	Reverse	GCCTGCAGACTTCAGTGG

*Il6* (mouse) primer sets were used in a previous study [44]

### RNA isolation and quantification of gene expression

Total RNA was extracted from fibroblasts using an RNeasy Mini Kit (Qiagen) according to the manufacturer’s instructions. To remove DNA, a gDNA Eliminator Spin Column (Qiagen) was used as described in the manual. Reverse transcription reactions were performed using 30–300 ng of RNA as template with ReverTra Ace qPCR RT Master Mix (TOYOBO). Reverse-transcribed cDNA was subjected to PCR and quantitative PCR (qPCR). PCR and qPCR were performed using TaKaRa Ex Taq Hot Start Version (TAKARA) and THUNDERBIRD SYBR qPCR Mix (TOYOBO), respectively. The primers used for PCR and qPCR are provided in Table 1.

### Necroptosis analysis

Mouse or DMR primary fibroblasts or SVER cells were seeded at a density of 1 × 10^4^ cells per well in 24-well plates and incubated for 24 h, and then the following reagents were added: murine TNFα (PeproTech, 50 ng/mL), z-VAD-fmk (Abcam, 20 µM), CHX (FUJIFILM, WAKO; 1 µg/mL), and Nec-1 (Sigma-Aldrich, 20 µM). After 24 h, cell death was analyzed by PI/Hoechst staining as described above.

### Statistical analysis

GraphPad Prism (GraphPad ver.7) was used for statistical analysis. Two groups were compared using the unpaired *t*-test. For multiple comparisons, the data were analyzed using a one-way analysis of variance followed by Dunnett’s multiple comparisons test. *P*-values < 0.05 were considered statistically significant. The sample sizes and numbers of replicates (at least three individuals or independent experiments) are described in the figure legends.

## Results

### Isolation and culture of fibroblasts from DMR skin

To isolate DMR skin fibroblasts, skin pieces from 1- to 3-year-old adult DMRs (Fig. 1a) were collected under anesthesia and cultured in fibroblast culture medium supplemented with 15% FBS, as previously described for NMR fibroblast culture [45]. The core body temperature of DMRs is about 35 °C [5, 9]; therefore, their skin pieces were cultured at this temperature. Skin fibroblasts from NMRs, which are closely related to DMRs, have been reported to be difficult to maintain under normoxic conditions and are typically cultured in 3–5% O_2_ [6, 37, 40]. Therefore, we conducted the primary culture of DMR skin pieces in 5% O_2_. After 9 days, we observed growth of fibroblasts from the skin fragments (Fig. 1b, c). These cells were harvested by trypsinization after 20–26 days of cultivation, frozen, and used for subsequent experiments (Fig. 1d).

### Determination of the optimal conditions for DMR fibroblast culture

**Fig. 2:**
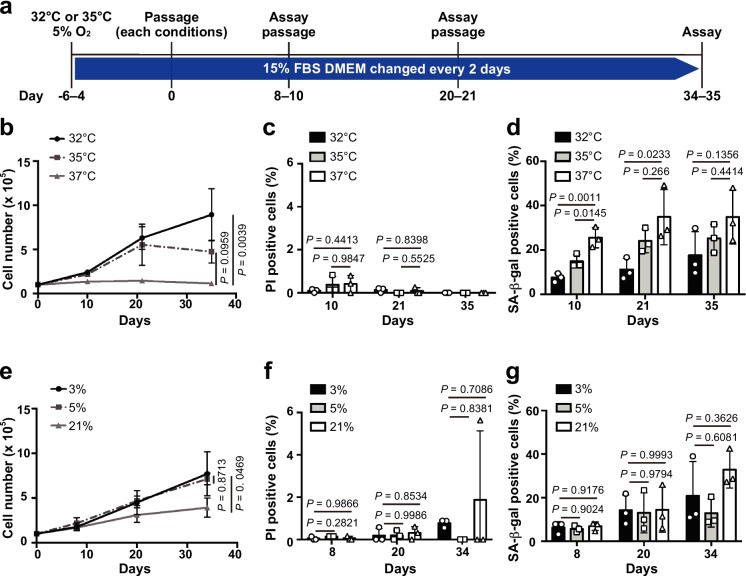
Determination of the optimal conditions for Damaraland mole-rat (DMR) fibroblast culture. **a** Timeline of DMR fibroblast culture for analysis of the optimal culture conditions. Preculture was performed at 35 °C in **b**–**d** and 32 °C in **e**–**g**. **b** Cell proliferation at each temperature. **c** Cell death at each temperature. **d** Cellular senescence at each temperature. **e** Cell proliferation at each O_2_ level. **f** Cell death at each O_2_ level. **g** Cellular senescence at each O_2_ level. Data are presented as the mean ± standard deviation of *n* = 3 biological replicates. A one-way analysis of variance with Dunnett’s multiple comparisons test versus 37 °C or 3% O_2_ was performed in **b**–**g**. In **c**, all values on day 35 were 0% and therefore these data could not be included in statistical analysis

In contrast with fibroblasts of mice, rats, and humans, which have an optimum culture temperature of 37 °C, fibroblasts of NMRs are generally cultured at a temperature approximating their body temperature of 32 °C [48] although some studies have successfully maintained NMR fibroblasts at 35 °C [49], a temperature more in keeping with that of pregnant NMRs [50] and more similar to captive core body temperature of DMRs [9, 18]. To determine the optimum temperature for culturing DMR fibroblasts, we compared their growth rate, death, and senescence at three temperatures: 32 °C, 35 °C, and 37 °C (Fig. 2a). Unexpectedly, cells proliferated most actively at 32 °C, whereas no cell proliferation was observed at 37 °C (Fig. 2b). At 35 °C, which is the core body temperature of DMRs, cell proliferation slowed down from 21 days after the start of experiments. The percentage of dead cells did not significantly differ between the temperatures (Fig. 2c). The percentage of SA-β-Gal-positive senescent cells was lowest at 32 °C, highest at 37 °C, and intermediate at 35 °C (Fig. 2d). These data collectively suggest that 32 °C rather than 35 °C is the optimum temperature for DMR fibroblast culture.

To investigate the impact of different oxygen levels on DMR fibroblasts, we cultured these cells in 3%, 5%, or 21% O_2_. Cell proliferation was slowest in 21% O_2_ and did not significantly differ between 3 and 5% O_2_ (Fig. 2e). While there was large variance, the percentage of cell death and senescent cells tended to increase with the number of days in culture, particularly for senescent cells at 21% O_2_ (Fig. 2f, g). These findings indicate that an atmospheric 21% O_2_ level is not suitable for DMR fibroblast culture similar to NMR fibroblast culture.

In summary, our results indicate that a temperature of 32 °C and a 3–5% O_2_ level are the optimal conditions for the culture of DMR skin fibroblasts. Under these conditions, cells could be cultured up to at least passage 10. We also confirmed that DMR skin fibroblasts could be isolated from the skin pieces at 32 °C in 5% O_2_.

### Generation and characterization of immortalized DMR fibroblasts by SV40ER transduction

**Fig. 3:**
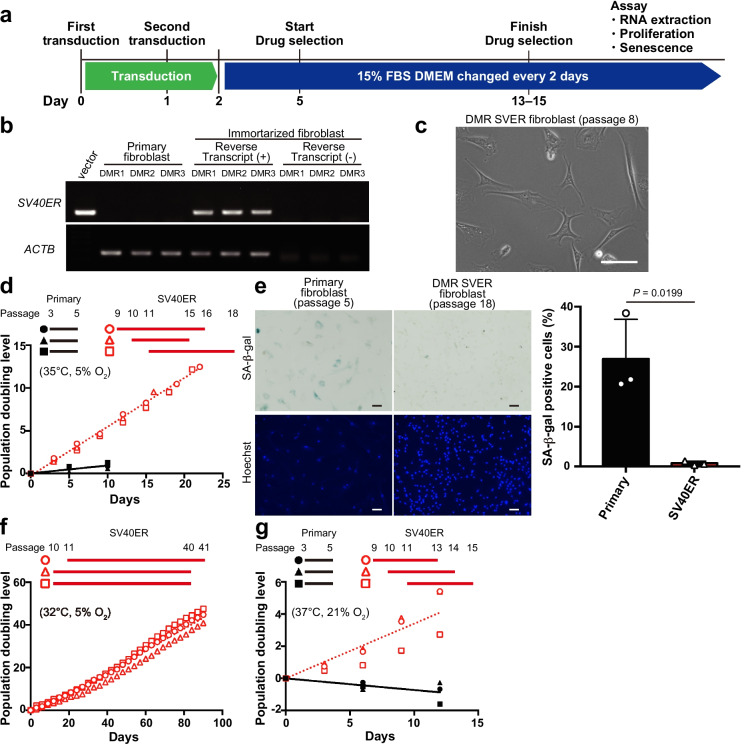
Generation and characterization of immortalized Damaraland mole-rat (DMR) fibroblasts by SV40 early region (SV40ER) transduction. **a** Timeline of generating immortalized DMR fibroblasts. **b** SV40ER expression in immortalized DMR fibroblasts. The pCSII-EF-SV40ER-TK-hyg vector was used as the positive control and reverse transcript (−) was used as the negative control. **c** Image of immortalized DMR fibroblasts. Scale bar: 100 µm. **d** Comparison of proliferation between SV40ER and primary cells at 35 °C in 5% O_2_. **e** Images and percentages of senescent primary and immortalized DMR fibroblasts. Primary fibroblasts at passage 5–6 and DMR SVER fibroblasts at passage 15–18 were used. Scale bar: 100 µm. **f** Cell proliferation of DMR SVER cells at 32 °C in 5% O_2_. **g** Comparison of proliferation between SV40ER and primary cells at 37 °C in 21% O_2_. In **d**, **f**, and **g**, the values of three biological replicates were shown. Lines were fitted to the data by simple linear regression. In **e**, data are presented as the mean ± standard deviation of *n* = 3 biological replicates. The unpaired *t*-test was performed

Long-term maintenance of DMR fibroblasts poses challenges because cellular senescence increases and the proliferation rate decreases with passaging (Fig. 2d, g). To overcome this limitation, we aimed to generate immortalized DMR fibroblasts by performing lentiviral transduction of SV40ER (DMR SVER cells, Fig. 3a). After selection with hygromycin B for 8–10 days, DMR SVER cells were expanded, frozen, and used for subsequent analyses. RT-PCR analysis confirmed that expression of SV40ER was high in DMR fibroblasts at 4 weeks after transduction (Fig. 3b). Transduction of SV40ER made the cells more spindle-shaped and smaller (Fig. 3c). DMR SVER cells proliferated more vigorously and exhibited a significant decrease in senescence than primary fibroblasts at 35 °C in 5% O_2_ (Fig. 3d, e). The proliferation ability of DMR SVER cells was stable for over 30 sequential passages (Fig. 3f). Importantly, DMR SVER cells proliferated at 37 °C in 21% O_2_, where primary fibroblasts could not (Fig. 3g). Thus, transduction of SV40ER enabled the generation of immortalized DMR fibroblasts.

### Resistance of DMR fibroblasts to various cytotoxic agents

**Fig. 4:**
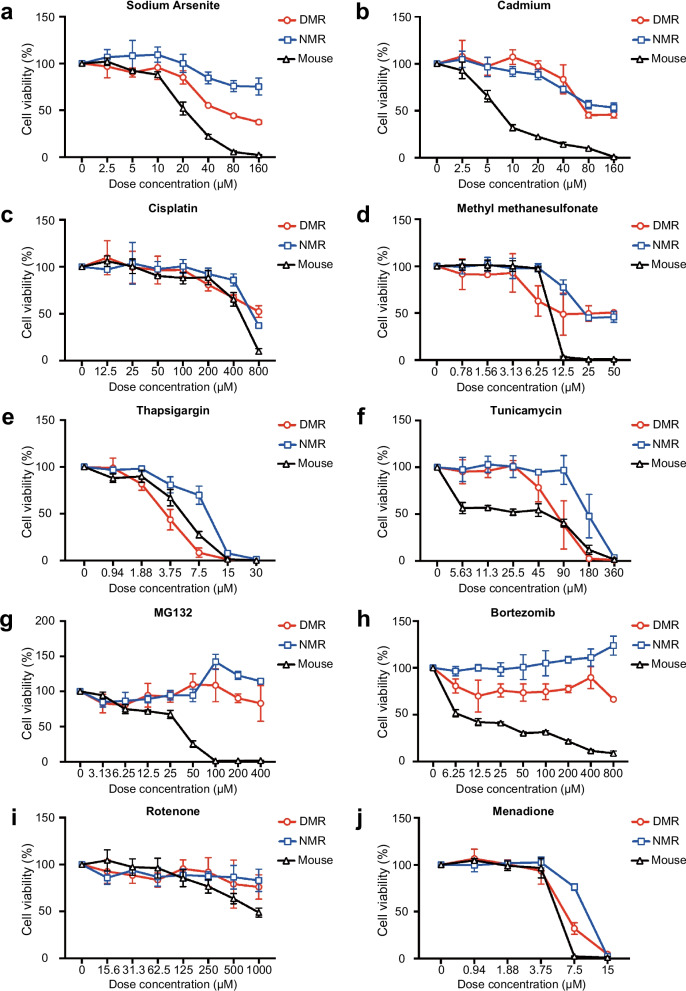
Resistance of Damaraland mole-rat (DMR) fibroblasts to various cytotoxic agents. Cell viability of DMR, naked mole-rat (NMR), and mouse skin fibroblast exposed to sodium arsenite (**a**), cadmium (**b**), cisplatin (**c**), methyl methanesulfonate (**d**), thapsigargin (**e**), tunicamycin (**f**), MG132 (**g**), bortezomib (**h**), rotenone (**i**), and menadione (**j**). Data are presented as the mean ± standard deviation of *n* = 3 technical replicates

Fibroblasts of DMRs, mice, and NMRs were exposed for 2 h to heavy metals (arsenic, cadmium), DNA damaging agents (cisplatin, methyl methanesulfonate [MMS]), endoplasmic reticulum (ER) stressors (thapsigargin, tunicamycin), protein degradation inhibitors (MG132, bortezomib), and mitochondrial toxins (rotenone, menadione) (Fig. 4). Twenty-four hours later, cell viability was measured. These data showed that when the cells of the different species were housed at their optimal body temperature with all other experimental conditions and doses of toxins being identical, the cells of both NMR and DMR showed greater resistance to heavy metals, DNA damaging agents, protein degradation blockers, and mitochondrial inhibitors, but not to ER stressors, than those of the mouse (Fig. 4). The DMR cells were generally slightly more sensitive to toxins than the NMR cells.

### Loss of necroptosis-inducing ability in DMRs

**Fig. 5:**
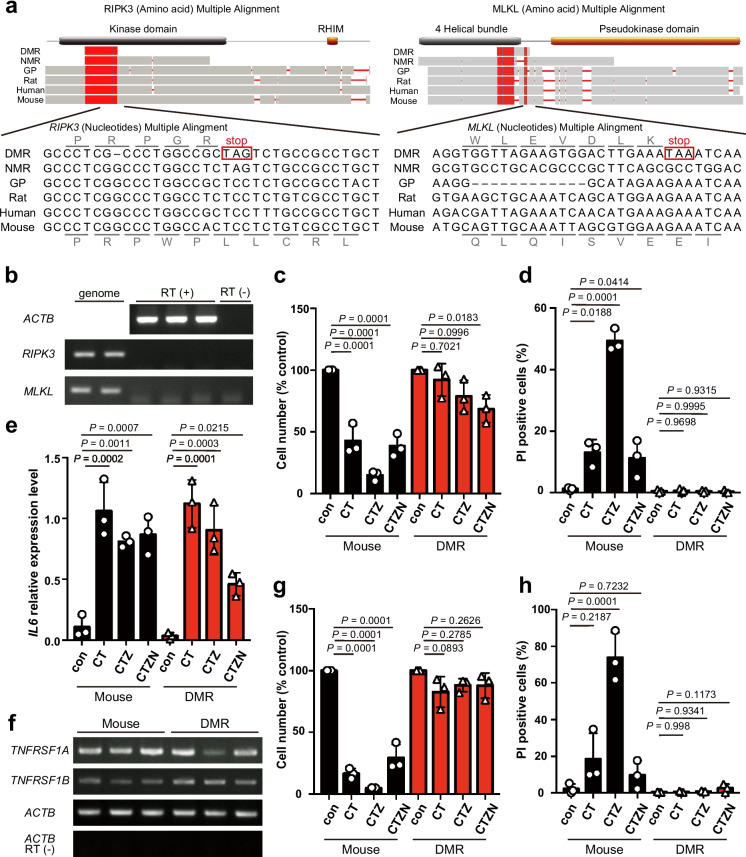
Loss of necroptosis-inducing ability in Damaraland mole-rats (DMRs). **a** Multiple alignments of receptor-interacting protein kinase 3 (*RIPK3*) and mixed lineage kinase domain-like (*MLKL*) sequences from the DMR, naked mole-rat (NMR), guinea pig (GP), rat, human, and mouse. Premature stop codons in the DMR sequence are boxed. Reading frames for the DMR and mouse sequences are indicated. The functional domains are shown above the alignments. **b** Semi-quantitative RT-PCR analysis of expression of *RIPK3*, *MLKL*, and *ACTB* using cDNA from DMR skin fibroblasts. Genomic DNA was used as the positive control and reverse transcript (RT) (−) was used as the negative control. Cell number (**c**) and cell death (**d**) of DMR and mouse primary fibroblasts treated with a combination of TNFα (T), cycloheximide (C), z-VAD-fmk (Z), or Nec-1 (N). **e** Relative expression level of interleukin-6 (*IL6*) mRNA in DMR and mouse primary fibroblasts treated with a combination of TNFα (T), cycloheximide (C), z-VAD-fmk (Z), or Nec-1 (N) for 6 h. **f** Semi-quantitative RT-PCR analysis of expression of TNF receptor superfamily member 1A (*TNFRSF1A*, encoding TNFR1) and 1B (*TNFRSF1B*, encoding TNFR2) mRNA in DMR and mouse primary fibroblasts. *ACTB* was used as the positive control and reverse transcript (RT) (−) was used as the negative control. Cell number (**g**) and cell death (**h**) of DMR and mouse SVER cells treated with a combination of TNFα (T), cycloheximide (C), z-VAD-fmk (Z), or Nec-1 (N). Data are presented as the mean ± standard deviation of *n* = 3 biological replicates. A one-way analysis of variance with Dunnett’s multiple comparisons test versus the untreated control (con) was performed in **c**–**e**, **g**, and **h**

We previously reported that NMRs have lost the ability to induce necroptosis due to loss-of-function mutations in the master regulator genes of necroptosis, *RIPK3* and *MLKL*. This loss likely contributes to carcinogenesis resistance of NMRs by attenuating tissue inflammatory responses associated with cancer promotion [44]. It has been predicted that the DMR genome harbors premature stop codons in the coding sequences of the *RIPK3* and *MLKL* genes [47]. We attempted to confirm these mutations in *RIPK3* and *MLKL* by cloning and Sanger sequencing of the genomic DNA of these genes from DMR skin. The position of the premature stop codon was consistent with that previously predicted for *MLKL*, but occurred upstream of the expected position for *RIPK3* [47]. These premature stop codons were located more upstream than in NMRs, and the N-terminal sequences of the genes were not annotated in the DMR genome (Fig. 5a). These premature stop codons and sequence changes resulted in loss of the majority of the kinase domain and all of the RHIM domain in DMR RIPK3 and complete loss of the pseudokinase domain in DMR MLKL, which are functionally essential to induce necroptosis in other mammalian species [51]. Moreover, expression of *RIPK3* and *MLKL* was not detected in DMR fibroblasts by RT-PCR (Fig. 5b).

Next, we investigated whether the necroptosis-inducing ability is lost in DMRs. We experimentally induced RIPK1-mediated necroptosis in DMR fibroblasts by treating them with TNFα, cycloheximide (CHX), and z-VAD-fmk (a caspase inhibitor). In mouse fibroblasts, this treatment significantly increased cell death, which was suppressed by addition of Necrostatin-1 (a RIPK1 inhibitor) (Fig. 5c, d), confirming the induction of necroptosis as previously reported [52]. These treatments increased TNFα-induced *Il6* expression in mouse fibroblasts (Fig. 5e). In DMR cells, upregulation of *IL6* by each treatment, along with expression of TNFR1 and TNFR2, suggests the activation of TNF signaling (Fig. 5e, f). However, cell death was not significantly increased following TNFα + CHX or TNFα + CHX + z-VAD treatment (Fig. 5c, d), similar to the response of NMR fibroblasts we previously reported [44]. RIPK3 is important for the induction of both necroptosis and TNF-induced apoptosis mediated by RIPK1 in mice [53]. Therefore, these data indicate that DMRs, like their close relative NMRs, have likely lost the ability to induce TNFα-induced necroptosis and apoptosis due to loss-of-function mutations in the *RIPK3* and *MLKL* genes. Necroptosis resistance was also observed in DMR SVER cells (Fig. 5g, h).

## Discussion

In this study, we optimized the culture conditions for DMR primary fibroblasts, and generated immortalized DMR cells transduced with SV40ER. Using these cultured cells, we firstly showed DMR cells were more resistant than those of mice to most cytotoxins and demonstrated that DMRs have likely lost the ability to induce necroptosis due to loss-of-function mutations in the necroptosis master regulators *RIPK3* and *MLKL*.

We examined the culture conditions for primary DMR fibroblasts, focusing on temperature and oxygen levels, based on the culture medium used for NMR cells (Fig. 2). Unexpectedly, our results indicated that the optimal temperature for DMR fibroblast culture is 32 °C, which is significantly lower than their captive core body temperature of 35 °C. This discrepancy may be linked to pronounced seasonal fluctuation in the minimum body temperature of DMRs, which can drop to around 32 °C in winter and rise to around 34 °C in summer [9]. Regarding O_2_ conditions, a 3–5% O_2_ level was more suitable for DMR fibroblasts, although substantial individual variation was observed in cell death and cellular senescence. This is in keeping with the general physiological partial pressure of oxygen in somatic tissues other than the lung in mammals. Parrinello et al. reported that mouse fibroblasts suffer increased oxidative damage and a shortened replicative lifespan when cultured under 20% O_2_ compared with 3% O_2_ [54]. Compared with mouse fibroblasts, DMR fibroblasts may be more sensitive to normoxia as a result of adaptation to the hypoxic subterranean habitat, similar to NMR fibroblasts. Investigation of the composition of the dish coating and culture medium may further improve DMR fibroblast culture. In addition, due to the limited number of DMR individuals, experiments in this study were conducted with three biological replicates. However, the optimal culture conditions should be verified with a larger sample size in future studies.

Given that the core body temperature of DMRs is significantly lower than those of mice and humans, the standard 37 °C incubation conditions, which are suitable for many mammals, might be detrimental to DMR cells. Indeed, primary DMR fibroblasts rapidly stopped proliferating and exhibited increased SA-β-Gal activity at 37 °C (Fig. 2b, d). However, DMR SVER cells could proliferate at 37 °C under normoxia, whereas primary DMR fibroblasts normally could not (Fig. 3g). Furthermore, DMR SVER cells demonstrated significant suppression of senescence and maintained their proliferation capacity even after 40 passages (Fig. 3e, f). In a previous paper, NMR fibroblasts transfected with the SV40 large T-antigen and human Ras harboring the G12V substitution proliferated more rapidly than primary NMR fibroblasts, without a significant difference in translational fidelity [41]. The proliferation capacity of DMR SVER cells at 37 °C under normoxia enables their culture under the same conditions as those used for human and mouse cells. Thus, although the transduction of SVER alters some cellular characteristics, including temperature or O_2_ sensitivity, DMR SVER cells are accessible and suitable for certain comparative studies with other mammalian cells, such as necroptosis assays (Fig. 5g, h).

Cytotoxic stress resistance is considered an important predictor of species lifespan and healthspan [48]. Not only will stress resistance result in augmented resilience against environmental toxins, but it will also contribute to a more stable proteome and genome, resulting in the long-term maintenance of cellular homeostasis. Stress resistance may also protect against inflammation and other symptoms associated with chronic age-related diseases, e.g., neurodegeneration and cancer. Primary DMR fibroblasts were more resistant to toxic cell insults including heavy metals, DNA-damaging agents, proteasome inhibitors, and oxidative stressors when cultured under their body temperature conditions (Fig. 4). The exception was the response of DMR cells to the ER stressor (thapsigargin and tunicamycin) that blocks the sarco/endoplasmic reticulum (ER) Ca^2+^-ATPase (SERCA), disrupting Ca^2+^ homeostasis, and inhibits N-glycosylation resulting in protein misfolding, impeding the transport of proteins from the ER and inducing cell death. Similar resistance to various cytotoxic drugs has been reported for NMR fibroblasts as well as those from long-lived mutant mice [39, 55, 56], bats [57], and birds [58]. This type of cytoprotection moves beyond simply neutralization of oxidative stress, for DMRs reportedly do not exhibit superior antioxidant profiles [35]. Moreover, this resilience is also evident in blood vessels against other exogenous chemical stressors and how the species responds to mitigate the damage impact and in the case of DNA damaging agents may reflect DMR resistance to DNA fragmentation, enhanced DNA repair pathways, and resistance to caspase activation [59]. Transcriptomic analyses support the premise of augmented DNA repair pathways with higher expression of genes associated with the DNA damage response and repair under normoxic conditions. Additionally, the inactivation of Fas-activated serine/threonine kinase (FASTK), which contributes to the regulation of Fas-mediated apoptosis, was also identified [21].

In this study, we found that DMRs have likely lost the necroptosis-inducing ability due to loss-of-function mutations in the *RIPK3* and *MLKL* genes, similar to the loss in NMRs we previously reported [44] (Fig. 5). Notably, DMRs and NMRs harbor mutations at different positions that cause premature stop codons in *RIPK3* and *MLKL*, suggesting these mutations were obtained independently of each other. These findings mean this trait is conserved between these two African mole-rat species and has been subject to positive selection. To clarify the process by which defects of these necroptosis genes arose, it is necessary to obtain more detailed genomic information of DMRs and to examine its conservation in other African mole-rat species. Our previous study demonstrated that the loss of necroptosis-inducing ability likely contributes to the carcinogenesis resistance through attenuated tissue inflammatory responses of NMRs [44]. In pancreatic ductal adenocarcinoma in mice, necroptosis induces an immunosuppressive tumor microenvironment via CXCL1 expression, contributing to cancer progression [60]. It is possible that DMRs exhibit cancer resistance phenotypes similar to NMRs. Necroptosis may not only contribute to the promotion of tumor growth but also to the exacerbation of other pathological conditions associated with chronic inflammation, including ischemia–reperfusion injury, atherosclerosis, neurodegenerative diseases, and inflammatory bowel diseases [61, 62]. Future studies are crucial to verify inflammatory responses and resistance to inflammation-related diseases, including cancer, in DMRs.

Previous studies suggested that high-molecular-mass hyaluronan is produced in DMRs as well as NMRs, which may contribute to cancer and aging resistance [38, 63]. Various cellular traits, such as induction of senescent cell death [40], loss-of-function of *ERAS* and a unique type of cellular senescence dependent on suppression of the tumor suppressor *ARF* [45], resistance to DNA damage [42], hypersensitive contact inhibition [64], and high translational fidelity [41], are suggested to potentially contribute to longevity, delayed aging, and cancer resistance in NMRs. By verifying whether these characteristics are preserved in DMR cells, we can gain insights into the evolutionarily conserved cellular mechanisms associated with “healthy aging” in African mole-rat species, as well as into the species-specific acquired traits. Our study, which optimized the culture conditions for primary DMR fibroblasts and established immortalized DMR fibroblasts, will help to improve in vitro studies of DMRs. Studies comparing the traits of NMR and DMR fibroblasts have the potential to significantly deepen our understanding of the mechanisms underlying the unique traits of African mole-rat species, including longevity and hypoxia tolerance.

## Data Availability

The datasets utilized in this study are available from the corresponding author upon request.

## References

[1] Bennett NC, Jarvis JUM. The social structure and reproductive biology of colonies of the mole-rat, Cryptomys damarensis (Rodentia, Bathyergidae). J Mammal. 1988;69:293–302. 10.2307/1381379.

[2] Jarvis JUM, Bennett NC. Eusociality has evolved independently in two genera of bathyergid mole-rats - but occurs in no other subterranean mammal. Behav Ecol Sociobiol. 1993;33:253–60 (https://www.jstor.org/stable/4600876).

[3] Bennett NC, Jarvis JUM. Cryptomys damarensis. Mammalian species. 2004;756:1–5. 10.1644/756.

[4] McNab BK. The metabolism of fossorial rodents: a study of convergence. Ecology. 1966;47:712–33. 10.2307/1934259.

[5] Lovegrove BG. The metabolism of social subterranean rodents: adaptation to aridity. Oecologia. 1986;69:551–5. 10.1007/BF00410361. 28311614 10.1007/BF00410361

[6] Yap KN, Wong HS, Ramanathan C, Rodriguez-Wagner CA, Roberts MD, Freeman DA, Buffenstein R, Zhang Y. Naked mole-rat and Damaraland mole-rat exhibit lower respiration in mitochondria, cellular and organismal levels. Biochim Biophys Acta Bioenerg. 2022;1863: 148582. 10.1016/j.bbabio.2022.148582. 35667393 10.1016/j.bbabio.2022.148582 PMC10316699

[7] Scantlebury M, Speakman JR, Oosthuizen MK, Roper TJ, Bennett NC. Energetics reveals physiologically distinct castes in a eusocial mammal. Nature. 2006;440:795–7. 10.1038/nature04578. 16598257 10.1038/nature04578

[8] Buffenstein R, Craft W. The idiosyncratic physiological traits of the naked mole-rat; a resilient animal model of aging, longevity, and healthspan BT - the extraordinary biology of the naked mole-rat. In: Buffenstein R, Park TJ, Holmes MM, editors. Cham: Springer International Publishing; 2021. p. 221–54. 10.1007/978-3-030-65943-1_8 10.1007/978-3-030-65943-1_8 34424518

[9] Streicher S, Boyles JG, Oosthuizen MK, Bennett NC. Body temperature patterns and rhythmicity in free-ranging subterranean Damaraland mole-rats. Fukomys damarensis PLoS One. 2011;6: e26346. 10.1371/journal.pone.0026346. 22028861 10.1371/journal.pone.0026346 PMC3196572

[10] Buffenstein R. Ecophysiological responses to a subterranean habitat; a Bathyergid perspective. Mammalia. 1996;60:591–606. 10.1515/mamm.1996.60.4.591.

[11] Zhang SY, Pamenter ME. Ventilatory, metabolic, and thermoregulatory responses of Damaraland mole rats to acute and chronic hypoxia. J Comp Physiol B. 2019;189:319–34. 10.1007/s00360-019-01206-y. 30725174 10.1007/s00360-019-01206-y

[12] Ivy CM, Sprenger RJ, Bennett NC, van Jaarsveld B, Hart DW, Kirby AM, Yaghoubi D, Storey KB, Milsom WK, Pamenter ME. The hypoxia tolerance of eight related African mole-rat species rivals that of naked mole-rats, despite divergent ventilatory and metabolic strategies in severe hypoxia. Acta Physiol. 2020;228: e13436. 10.1111/apha.13436. 10.1111/apha.13436 31885213

[13] Buffenstein R, Skinner DC, Yahav S, Moodley GP, Cavaleros M, Zachen D, Ross FP, Pettifor JM. Effect of oral cholecalciferol supplementation at physiological and supraphysiological doses in naturally vitamin D3-deficient subterranean Damara mole rats (Cryptomys damarensis). J Endocrinol. 1991;131:197–202. 10.1677/joe.0.1310197. 1660517 10.1677/joe.0.1310197

[14] Pitcher T, Buffenstein R, Keegan JD, Moodley GP, Yahav S. Dietary calcium content, calcium balance and mode of uptake in a subterranean mammal, the Damara mole-rat. J Nutr. 1992;122:108–14. 10.1093/jn/122.1.108. 1729458 10.1093/jn/122.1.108

[15] Lewis KN, Buffenstein R. Chapter 6 - The naked mole-rat: a resilient rodent model of aging, longevity, and healthspan. In: Kaeberlein MR, Martin GMBT-H of the B of A (Eighth E, editors. San Diego: Academic Press; 2016 179–204. 10.1016/B978-0-12-411596-5.00006-X

[16] Smith ESJ, Park TJ, Lewin GR. Independent evolution of pain insensitivity in African mole-rats: origins and mechanisms. J Comp Physiol A Neuroethol Sens Neural Behav Physiol. 2020;206:313–25. 10.1007/s00359-020-01414-w. 32206859 10.1007/s00359-020-01414-w PMC7192887

[17] Wong HS, Freeman DA, Zhang Y. Not just a cousin of the naked mole-rat: Damaraland mole-rats offer unique insights into biomedicine. Comp Biochem Physiol B Biochem Mol Biol. 2022;262: 110772. 10.1016/j.cbpb.2022.110772. 35710053 10.1016/j.cbpb.2022.110772 PMC10155858

[18] Buffenstein R, Smith M, Amoroso VG, Patel TT, Ross M, Bassanpal S, Park TJ, Delaney MA, Adams CR, Arroyo J, Fortman J. A new laboratory research model: the Damaraland mole-rat and its managed care. J Am Assoc Lab Anim Sci. 2024. 10.30802/AALAS-JAALAS-24-052 10.30802/AALAS-JAALAS-24-052 PMC11645877 39179367

[19] Hulbert AJ, Pamplona R, Buffenstein R, Buttemer WA. Life and death: metabolic rate, membrane composition, and life span of animals. Physiol Rev. 2007;87:1175–213. 10.1152/physrev.00047.2006. 17928583 10.1152/physrev.00047.2006

[20] Ruby JG, Smith M, Buffenstein R. Five years later, with double the demographic data, naked mole-rat mortality rates continue to defy Gompertzian laws by not increasing with age. Geroscience. 2024. 10.1007/s11357-024-01201-4. 38773057 10.1007/s11357-024-01201-4 PMC11336006

[21] Fang X, Seim I, Huang Z, Gerashchenko MV, Xiong Z, Turanov AA, Zhu Y, Lobanov AV, Fan D, Yim SH, Yao X, Ma S, Yang L, et al. Adaptations to a subterranean environment and longevity revealed by the analysis of mole rat genomes. Cell Rep. 2014;8:1354–64. 10.1016/j.celrep.2014.07.030. 25176646 10.1016/j.celrep.2014.07.030 PMC4350764

[22] Buffenstein R, Ruby JG. Opportunities for new insight into aging from the naked mole-rat and other non-traditional models. Nat Aging. 2021;1:3–4. 10.1038/s43587-020-00012-4. 37117998 10.1038/s43587-020-00012-4

[23] Oka K, Yamakawa M, Kawamura Y, Kutsukake N, Miura K. The naked mole-rat as a model for healthy aging. Annu Rev Anim Biosci. 2023;11:207–26. 10.1146/annurev-animal-050322-074744. 36318672 10.1146/annurev-animal-050322-074744

[24] Ruby JG, Smith M, Buffenstein R. Naked mole-rat mortality rates defy Gompertzian laws by not increasing with age. Elife. 2018;7: e31157. 10.7554/eLife.31157. 29364116 10.7554/eLife.31157 PMC5783610

[25] O’Connor TP, Lee A, Jarvis JUM, Buffenstein R. Prolonged longevity in naked mole-rats: age-related changes in metabolism, body composition and gastrointestinal function. Comp Biochem Physiol A Mol Integr Physiol. 2002;133:835–42. 10.1016/s1095-6433(02)00198-8. 12443939 10.1016/s1095-6433(02)00198-8

[26] Buffenstein R. Negligible senescence in the longest living rodent, the naked mole-rat: insights from a successfully aging species. J Comp Physiol B. 2008;178:439–45. 10.1007/s00360-007-0237-5. 18180931 10.1007/s00360-007-0237-5

[27] Can E, Smith M, Boukens BJ, Coronel R, Buffenstein R, Riegler J. Naked mole-rats maintain cardiac function and body composition well into their fourth decade of life. Geroscience. 2022;44:731–46. 10.1007/s11357-022-00522-6. 35107705 10.1007/s11357-022-00522-6 PMC9135933

[28] Edrey YH, Park TJ, Kang H, Biney A, Buffenstein R. Endocrine function and neurobiology of the longest-living rodent, the naked mole-rat. Exp Gerontol. 2011;46:116–23. 10.1016/j.exger.2010.09.005. 20888895 10.1016/j.exger.2010.09.005

[29] Voigt C, Bennett NC. Gene expression pattern of Kisspeptin and RFamide-related peptide (Rfrp) in the male Damaraland mole-rat hypothalamus. J Chem Neuroanat. 2021;118: 102039. 10.1016/j.jchemneu.2021.102039. 34655735 10.1016/j.jchemneu.2021.102039

[30] Hart DW, Bennett NC, Voigt C. Social stress is unlikely to play a major role in reproductive suppression of female subordinate naked mole-rats and Damaraland mole-rats. Biol Lett. 2022;18:20220292. 10.1098/rsbl.2022.0292. 36285462 10.1098/rsbl.2022.0292 PMC9597399

[31] Faulkes CG, Bennett NC. Social evolution in African mole-rats – a comparative overview BT - the extraordinary biology of the naked mole-rat. In: Buffenstein R, Park TJ, Holmes MM, editors. Cham: Springer International Publishing; 2021. p. 1–33. 10.1007/978-3-030-65943-1_1 10.1007/978-3-030-65943-1_1 34424511

[32] Houslay TM, Vullioud P, Zöttl M, Clutton-Brock TH. Benefits of cooperation in captive Damaraland mole-rats. Behav Ecol. 2020;31:711–8. 10.1093/beheco/araa015.

[33] Thorley J, Bensch HM, Finn K, Clutton-Brock T, Zöttl M. Damaraland mole-rats do not rely on helpers for reproduction or survival. Evol Lett. 2023;7:203–15. 10.1093/evlett/qrad023. 37475748 10.1093/evlett/qrad023 PMC10355180

[34] Lambert AJ, Boysen HM, Buckingham JA, Yang T, Podlutsky A, Austad SN, Kunz TH, Buffenstein R, Brand MD. Low rates of hydrogen peroxide production by isolated heart mitochondria associate with long maximum lifespan in vertebrate homeotherms. Aging Cell. 2007;6:607–18. 10.1111/j.1474-9726.2007.00312.x. 17596208 10.1111/j.1474-9726.2007.00312.x

[35] Schmidt CM, Blount JD, Bennett NC. Reproduction is associated with a tissue-dependent reduction of oxidative stress in eusocial female Damaraland mole-rats (Fukomys damarensis). PLoS ONE. 2014;9: e103286. 10.1371/journal.pone.0103286. 25068591 10.1371/journal.pone.0103286 PMC4113376

[36] Jacobs PJ, Hart DW, Bennett NC. Plasma oxidative stress in reproduction of two eusocial African mole-rat species, the naked mole-rat and the Damaraland mole-rat. Front Zool. 2021;18:45. 10.1186/s12983-021-00430-z. 34535150 10.1186/s12983-021-00430-z PMC8447654

[37] Zhao Y, Zheng Z, Zhang Z, Xu Y, Hillpot E, Lin YS, Zakusilo FT, Lu JY, Ablaeva J, Biashad SA, Miller RA, Nevo E, Seluanov A, et al. Evolution of high-molecular-mass hyaluronic acid is associated with subterranean lifestyle. Nat Commun. 2023;14:8054. 10.1038/s41467-023-43623-2. 38052795 10.1038/s41467-023-43623-2 PMC10698142

[38] Tian X, Azpurua J, Hine C, Vaidya A, Myakishev-Rempel M, Ablaeva J, Mao Z, Nevo E, Gorbunova V, Seluanov A. High-molecular-mass hyaluronan mediates the cancer resistance of the naked mole rat. Nature. 2013;499:346–9. 10.1038/nature12234. 23783513 10.1038/nature12234 PMC3720720

[39] Salmon AB, Sadighi Akha AA, Buffenstein R, Miller RA. Fibroblasts from naked mole-rats are resistant to multiple forms of cell injury, but sensitive to peroxide, ultraviolet light, and endoplasmic reticulum stress. J Gerontol A Biol Sci Med Sci. 2008;63:232–41. 10.1093/gerona/63.3.232. 18375872 10.1093/gerona/63.3.232 PMC2710579

[40] Kawamura Y, Oka K, Semba T, Takamori M, Sugiura Y, Yamasaki R, Suzuki Y, Chujo T, Nagase M, Oiwa Y, Fujioka S, Homma S, Yamamura Y, et al. Cellular senescence induction leads to progressive cell death via the INK4a-RB pathway in naked mole-rats. EMBO J. 2023;42:e111133. 10.15252/embj.2022111133. 37431790 10.15252/embj.2022111133 PMC10425838

[41] Azpurua J, Ke Z, Chen IX, Zhang Q, Ermolenko DN, Zhang ZD, Gorbunova V, Seluanov A. Naked mole-rat has increased translational fidelity compared with the mouse, as well as a unique 28S ribosomal RNA cleavage. Proc Natl Acad Sci U S A. 2013;110:17350–5. 10.1073/pnas.1313473110. 24082110 10.1073/pnas.1313473110 PMC3808608

[42] Tian X, Firsanov D, Zhang Z, Cheng Y, Luo L, Tombline G, Tan R, Simon M, Henderson S, Steffan J, Goldfarb A, Tam J, Zheng K, et al. SIRT6 is responsible for more efficient DNA double-strand break repair in long-lived species. Cell. 2019;177:622-638.e22. 10.1016/j.cell.2019.03.043. 31002797 10.1016/j.cell.2019.03.043 PMC6499390

[43] Evdokimov A, Kutuzov M, Petruseva I, Lukjanchikova N, Kashina E, Kolova E, Zemerova T, Romanenko S, Perelman P, Seluanov A, Gorbunova V, Graphodatsky A, Trifonov V, et al. Naked mole rat cells display more efficient excision repair than mouse cells. Aging. 2018;10:1454–73. 10.18632/aging.101482. 29930219 10.18632/aging.101482 PMC6046242

[44] Oka K, Fujioka S, Kawamura Y, Komohara Y, Chujo T, Sekiguchi K, Yamamura Y, Oiwa Y, Omamiuda-Ishikawa N, Komaki S, Sutoh Y, Sakurai S, Tomizawa K, et al. Resistance to chemical carcinogenesis induction via a dampened inflammatory response in naked mole-rats. Commun Biol. 2022;5:287. 10.1038/s42003-022-03241-y. 35354912 10.1038/s42003-022-03241-y PMC8967925

[45] Miyawaki S, Kawamura Y, Oiwa Y, Shimizu A, Hachiya T, Bono H, Koya I, Okada Y, Kimura T, Tsuchiya Y, Suzuki S, Onishi N, Kuzumaki N, et al. Tumour resistance in induced pluripotent stem cells derived from naked mole-rats. Nat Commun. 2016;7:11471. 10.1038/ncomms11471. 27161380 10.1038/ncomms11471 PMC4866046

[46] Yamaguchi S, Nohara S, Nishikawa Y, Suzuki Y, Kawamura Y, Miura K, Tomonaga K, Ueda K, Honda T. Characterization of an active LINE-1 in the naked mole-rat genome. Sci Rep. 2021;11:5725. 10.1038/s41598-021-84962-8. 33707548 10.1038/s41598-021-84962-8 PMC7952902

[47] Águeda-Pinto A, Alves LQ, Neves F, McFadden G, Jacobs BL, Castro LFC, Rahman MM, Esteves PJ. Convergent loss of the necroptosis pathway in disparate mammalian lineages shapes viruses countermeasures. Front Immunol. 2021;12: 747737. 10.3389/fimmu.2021.747737. 34539677 10.3389/fimmu.2021.747737 PMC8445033

[48] Lewis KN, Mele J, Hornsby PJ, Buffenstein R. Stress resistance in the naked mole-rat: the bare essentials - a mini-review. Gerontology. 2012;58:453–62. 10.1159/000335966. 22572398 10.1159/000335966 PMC4439786

[49] Liang S, Mele J, Wu Y, Buffenstein R, Hornsby PJ. Resistance to experimental tumorigenesis in cells of a long-lived mammal, the naked mole-rat (Heterocephalus glaber). Aging Cell. 2010;9:626–35. 10.1111/j.1474-9726.2010.00588.x. 20550519 10.1111/j.1474-9726.2010.00588.x PMC3743245

[50] Urison NT, Buffenstein RB. Metabolic and body temperature changes during pregnancy and lactation in the naked mole rat (Heterocephalus glaber). Physiol Zool. 1995;68:402–20. 10.1086/physzool.68.3.30163776.

[51] Dondelinger Y, Hulpiau P, Saeys Y, Bertrand MJM, Vandenabeele P. An evolutionary perspective on the necroptotic pathway. Trends Cell Biol. 2016;26:721–32. 10.1016/j.tcb.2016.06.004. 27368376 10.1016/j.tcb.2016.06.004

[52] Degterev A, Zhou W, Maki JL, Yuan J. Assays for necroptosis and activity of RIP kinases. Methods Enzymol. 2014;545:1–33. 10.1016/B978-0-12-801430-1.00001-9. 25065884 10.1016/B978-0-12-801430-1.00001-9

[53] Dondelinger Y, Aguileta MA, Goossens V, Dubuisson C, Grootjans S, Dejardin E, Vandenabeele P, Bertrand MJM. RIPK3 contributes to TNFR1-mediated RIPK1 kinase-dependent apoptosis in conditions of cIAP1/2 depletion or TAK1 kinase inhibition. Cell Death Differ. 2013;20:1381–92. 10.1038/cdd.2013.94. 23892367 10.1038/cdd.2013.94 PMC3770330

[54] Parrinello S, Samper E, Krtolica A, Goldstein J, Melov S, Campisi J. Oxygen sensitivity severely limits the replicative lifespan of murine fibroblasts. Nat Cell Biol. 2003;5:741–7. 10.1038/ncb1024. 12855956 10.1038/ncb1024 PMC4940195

[55] Salmon AB, Murakami S, Bartke A, Kopchick J, Yasumura K, Miller RA. Fibroblast cell lines from young adult mice of long-lived mutant strains are resistant to multiple forms of stress. Am J Physiol Endocrinol Metab. 2005;289:E23–9. 10.1152/ajpendo.00575.2004. 15701676 10.1152/ajpendo.00575.2004

[56] Harper JM, Salmon AB, Leiser SF, Galecki AT, Miller RA. Skin-derived fibroblasts from long-lived species are resistant to some, but not all, lethal stresses and to the mitochondrial inhibitor rotenone. Aging Cell. 2007;6:1–13. 10.1111/j.1474-9726.2006.00255.x. 17156084 10.1111/j.1474-9726.2006.00255.x PMC2766812

[57] Salmon AB, Leonard S, Masamsetti V, Pierce A, Podlutsky AJ, Podlutskaya N, Richardson A, Austad SN, Chaudhuri AR. The long lifespan of two bat species is correlated with resistance to protein oxidation and enhanced protein homeostasis. FASEB J. 2009;23:2317–26. 10.1096/fj.08-122523. 19244163 10.1096/fj.08-122523 PMC2704589

[58] Ogburn CE, Austad SN, Holmes DJ, Kiklevich JV, Gollahon K, Rabinovitch PS, Martin GM. Cultured renal epithelial cells from birds and mice: enhanced resistance of avian cells to oxidative stress and DNA damage. J Gerontol A Biol Sci Med Sci. 1998;53:B287–92. 10.1093/gerona/53a.4.b287. 18314559 10.1093/gerona/53a.4.b287

[59] Labinskyy N, Csiszar A, Orosz Z, Smith K, Rivera A, Buffenstein R, Ungvari Z. Comparison of endothelial function, O_2_^-.^ and H_2_O_2_ production, and vascular oxidative stress resistance between the longest-living rodent, the naked mole rat, and mice. Am J Physiol Heart Circ Physiol. 2006; 291: H2698–704 10.1152/ajpheart.00534.2006 10.1152/ajpheart.00534.2006 17090784

[60] Seifert L, Werba G, Tiwari S, Giao Ly NN, Alothman S, Alqunaibit D, Avanzi A, Barilla R, Daley D, Greco SH, Torres-Hernandez A, Pergamo M, Ochi A, et al. The necrosome promotes pancreatic oncogenesis via CXCL1 and Mincle-induced immune suppression. Nature. 2016;532:245–9. 10.1038/nature17403. 27049944 10.1038/nature17403 PMC4833566

[61] Weinlich R, Oberst A, Beere HM, Green DR. Necroptosis in development, inflammation and disease. Nat Rev Mol Cell Biol. 2017;18:127–36. 10.1038/nrm.2016.149. 27999438 10.1038/nrm.2016.149

[62] Dhuriya YK, Sharma D. Necroptosis: a regulated inflammatory mode of cell death. J Neuroinflammation. 2018;15:199. 10.1186/s12974-018-1235-0. 29980212 10.1186/s12974-018-1235-0 PMC6035417

[63] Zhang Z, Tian X, Lu JY, Boit K, Ablaeva J, Zakusilo FT, Emmrich S, Firsanov D, Rydkina E, Biashad SA, Lu Q, Tyshkovskiy A, Gladyshev VN, et al. Increased hyaluronan by naked mole-rat Has2 improves healthspan in mice. Nature. 2023;621:196–205. 10.1038/s41586-023-06463-0. 37612507 10.1038/s41586-023-06463-0 PMC10666664

[64] Seluanov A, Hine C, Azpurua J, Feigenson M, Bozzella M, Mao Z, Catania KC, Gorbunova V. Hypersensitivity to contact inhibition provides a clue to cancer resistance of naked mole-rat. Proc Natl Acad Sci U S A. 2009;106:19352–7. 10.1073/pnas.0905252106. 19858485 10.1073/pnas.0905252106 PMC2780760

